# Donor type and 3-month hospital readmission following kidney transplantation: results from the Netherlands organ transplant registry

**DOI:** 10.1186/s12882-021-02363-5

**Published:** 2021-04-27

**Authors:** Yiman Wang, Martin B. A. Heemskerk, Wieneke M. Michels, Aiko P. J. de Vries, Friedo W. Dekker, Yvette Meuleman

**Affiliations:** 1 Department of Clinical Epidemiology, Leiden University Medical Center, P.O. Box 9600, 2300 RC Leiden, The Netherlands; 2 Dutch Transplant Foundation, Leiden, The Netherlands; 3 Department of Internal Medicine, Division of Nephrology, Leiden University Medical Center, Leiden, The Netherlands; 4 Transplant Center, Leiden University Medical Center, Leiden, The Netherlands

**Keywords:** Kidney transplantation, Hospital readmission, Living donor, Deceased donor, Age

## Abstract

**Background:**

Hospital readmission after transplantation is common in kidney transplant recipients (KTRs). In this study, we aim to compare the risk of 3-month hospital readmission after kidney transplantation with different donor types in the overall population and in both young (< 65 years) and elderly (≥65 years) KTRs.

**Methods:**

We included all first-time adult KTRs from 2016 to 2018 in the Netherlands Organ Transplant Registry. Multivariable logistic regression models were used to estimate the effect while adjusting for baseline confounders.

**Results:**

Among 1917 KTRs, 615 (32.1%) had at least one hospital readmission. Living donor kidney transplantation (LDKT) recipients had an adjusted OR of 0.76 (95%CI, 0.61 to 0.96; *p* = 0.02) for hospital readmission compared to deceased donor kidney transplantation (DDKT) recipients. In the young and elderly, the adjusted ORs were 0.69 (95%CI, 0.52 to 0.90, *p* = 0.01) and 0.93 (95%CI, 0.62 to 1.39, *p* = 0.73) and did not differ significantly from each other (*p*-value for interaction = 0.38). In DDKT, the risk of hospital readmission is similar between recipients with donation after cardiac death (DCD) or brain death (DBD) and the risk was similar between the young and elderly.

**Conclusion:**

A lower risk of post-transplant 3-month hospital readmission was found in recipients after LDKT compared to DDKT, and this benefit of LDKT might be less dominant in elderly patients. In DDKT, having either DCD or DBD donors is not associated with post-transplant 3-month hospital readmission, regardless of age. Tailored patient management is needed for recipients with DDKT and elderly KTRs.

**Supplementary Information:**

The online version contains supplementary material available at 10.1186/s12882-021-02363-5.

## Introduction

Post-transplant hospital readmission poses a heavy burden on kidney transplant recipients (KTRs) as well as the medical system [1]. In the United States, approximately 30% of KTRs are readmitted within the first 30 days after transplantation, with an average medical cost of $11,719 for each hospital readmission [2]. Hospital admission by itself or as a marker for underlying medical conditions was also related to poor health-related quality of life [3, 4]. Furthermore, previous research revealed a strong association of early hospital readmission with death censored graft survival and patient survival in KTRs [5]. Recently, hospital (re) admission has been selected as a measure of disease burden by the International Consortium for Health Outcomes Measurement (ICHOM) working group in KTRs [6].

In the Netherlands, approximately 1000 kidney transplantations are performed annually [7]. Besides living donor kidney transplantation (LDKT), one-third of the patients on the waiting list accepted deceased donor kidney transplantation (DDKT) with donation either after cardiac death (DCD) or after brain death (DBD) [8]. From 2000 to 2017, 43% of DDKT within the Netherlands were performed with so-called controlled-DCD grafts (i.e. a graft recovered after withdrawing life-supporting treatment), which is among the highest percentage globally [9]. Previous studies showed that DCD donation could lead to acceptable proportions of graft and patient survival in spite of more risk of graft failure than DBD donation [9, 10]. It is also of considerable clinical interest to understand whether kidney transplantation with a specific donor type incurs a higher risk of post-transplant readmission. Such knowledge provides an opportunity to identify high-risk patients and allows an early treatment adjustment to reduce unnecessary hospital readmission and the overall medical cost in this population.

A few studies, mainly in the kidney transplant population of the United States, have investigated the association between donor type and hospital readmission [1, 2, 11, 12]. However, their findings might not be generalizable to other countries because of differences in organ acceptance policy and health care delivery system. Using data from the Dutch national registry, we investigated whether donor types (i.e. the living donor versus the deceased donor and the DBD donor versus the DCD donor) could be a risk factor for post-transplant 3-month hospital readmission in Dutch KTRs. With the growing geriatric population accepted for kidney transplantation, we also investigated if this relationship differs in young (< 65 years) and elderly (≥65 years) KTRs.

## Methods

Reporting of this registry study followed the STrengthening the Reporting of OBservational studies in Epidemiology (STROBE) guideline [13].

### Study design and study population

**Fig. 1:**
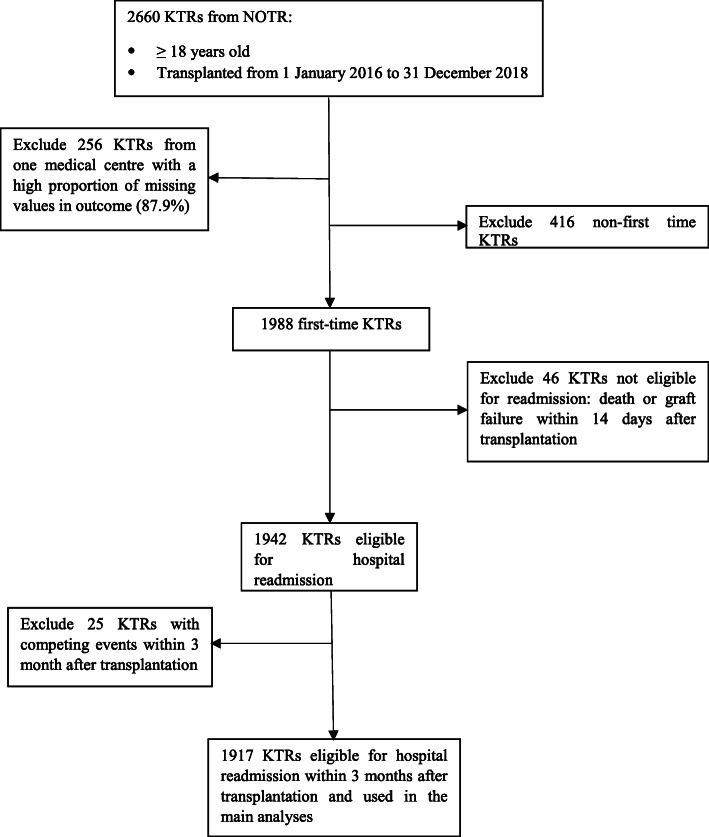
Flow chart for the patients included in this study

This study was conducted with data from the Netherlands Organ Transplant Registry (NOTR), a mandatory registry for kidney transplantation across all (eight) university medical centres within the Netherlands. The demographic and baseline clinical characteristics of both KTRs and donors are collected in the registry database. The registry follow-up starts at 3 months after kidney transplantation and annually thereafter. The follow-up ends when KTRs experience death or graft failure. In 2016, the NOTR began to include the number of hospital readmissions within the first 3 months after kidney transplantation into the database as a measurement of disease burden. KTRs in the Netherlands visit their transplant hospital regularly in the study period after kidney transplantation. The number of hospital readmissions was reported to the NOTR by health professionals from the participating medical centres based on patients’ medical records. In this analysis, we included all first-time KTRs over 18 years old transplanted from 1 January 2016 to 31 December 2018 (Fig. 1). All included patients have given written informed consent for submission of their data by the attending physician or hospital to the NOTR for the use of scientific research. The institutional review board of the NOTR approved this study and our use of the de-identified data. The study was conducted in accordance with the national guidelines for medical scientific research [14].

### Variables: exposure, outcome, and covariates

Donor type, the exposure of this study, was divided into 4 categories: the living donor and the deceased donor, and the deceased donor was further divided into two categories, namely the DCD donor and the DBD donor. The study outcome was binary, defined as at least one post-transplant hospital readmission within the first 3 months. Potential baseline confounders were identified for each comparison using the causal directed acyclic graph (DAG) approach [15]. According to this approach, confounders from the same path can be controlled by adjusting for one of them. More detailed information about DAGs and their application in nephrology has been described elsewhere [16]. To sufficiently adjust for confounding in the analysis comparing living donors and deceased donors, we selected a minimum set of confounders according to the DAG approach were: recipient age, recipient sex, primary renal disease (PRD), comorbidities, socioeconomic status (SES), panel-reactive antibody (PRA), and medical centre (**Figure** **S1**). For the comparison between DBD and DCD donors, the confounders to be adjusted were: recipient age, PRD, comorbidities, PRA, human leucocyte antigen-ABDR mismatch, donor characteristics (age, sex, body mass index [BMI], hypertension, last serum creatinine before donation, and extended criteria deceased donor), dialysis vintage, and medical centre (**Figure** **S2**).

PRD was grouped into six categories: diabetes, glomerulonephritis, renal vascular disease, cystic kidney disease, other diseases, and unknown ontology [17]. Comorbidities were identified by a history of cardiac event, vascular event, cerebrovascular accident, and diabetes prior to transplantation. The Transplant PRA was grouped into three categories: < 5, 6–85%, and > 85% [18]. Dialysis vintage was calculated as the time from the first record of dialysis to kidney transplantation. The SES rank of KTRs was the rank of the postal code area where KTRs lived. The latter was reported by the Netherlands Institute for Social Research and was derived from several characteristics of the residents in the neighbourhood (i.e. education, income, and position on the labour market) [19]. The extended criteria deceased donor was defined as any deceased donor over the age of 60, or a donor over the age of 50 with two of the following: a history of high blood pressure, a creatinine greater than or equal to 133 umol/L, or death resulting from a cerebrovascular cause [20].

### Statistical analysis

Means with standard deviations (SD) were calculated for normally distributed continuous variables and medians with interquartile ranges (IQR) for the non-normally distributed. Categorical variables were presented as counts and percentages. Multivariable logistic regression models were used to estimate the odds ratio (OR) of hospital readmission in KTRs with different donor types while adjusting for potential baseline confounders. In all analyses, LDKT and DDKT were compared, and within the DDKT subgroup, DCD and DBD donors were compared. Patients who survived for at least 14 days without graft failure were assumed as discharged from the index hospitalization (i.e. the hospital stay for kidney transplantation) and were eligible for hospital readmission. Patients who experienced competing events (i.e. graft failure and death) in the first 3 months were excluded from the analysis. Additionally, a subgroup analysis was performed for pre-specified age strata (young < 65 years and elderly ≥65 years) and the modification effect by age was tested by means of an interaction term.

In the comparison between LDKT and DDKT, the proportion of missing data in potential baseline confounders varied from 0.1 to 7.5% and 18.8% of the data for hospital readmission was missing. In the comparison between DDKT with DBD or DCD donors, the proportion of missing data in potential baseline confounders varied from 0.3 to 8.4% and 13.1% of the data for hospital readmission was missing (Tables 1 and 2). Missing values were imputed with 10-fold multiple imputation to maintain power and reduce bias [21]. Variables imputed were assumed to be missing at random and included: comorbidities, donor-related factors (BMI and hypertension), HLA-ABDR Mismatch, Transplant PRA, and hospital readmission within the first 3 months after kidney transplantation. The imputation model consisted of all variables (i.e. exposure, outcome, and potential baseline confounders) used in the analysis and also auxiliary variables including ABO incompatibility, the first warm ischemia time (WIT1, defined as the time between ending the blood circulation of the graft and starting the cold perfusion), the second warm ischemia time (WIT2, defined as the time from placing the graft into patients till revascularization of the graft), cold ischemia time (CIT, defined as the time from the start of cold perfusion to the removal of the renal allograft from ice), creatinine level in the first seven days and 3 months after kidney transplantation, and occurrence of delayed graft function (DGF, defined as the need for dialysis within the first week after kidney transplantation). The demographic and clinical characteristics for included KTRs with or without missing values in the outcome variable were tabulated (**Table** **S1**).

**Table 1:** Baseline characteristics of kidney transplant recipients eligible for hospital readmission within 3 months after transplantation (*n* = 1917)

Characteristics	LDKT recipients	DDKT recipients		
	(*n* = 1163, 60.7%)	DBD (*n* = 281, 14.6%)	DCD (*n* = 473, 24.7%)	Total (*n* = 754, 39.3%)
**Age category, yr, n(%)**				
18 ~ 39	227 (19.5)	18 (6.4)	43 (9.1)	61 (8.1)
40 ~ 59	510 (43.9)	97 (34.5)	152 (32.1)	249 (33.0)
60 ~ 64	167 (14.4)	44 (15.7)	86 (18.2)	130 (17.2)
65~	259 (22.3)	122 (43.4)	192 (40.6)	314 (41.6)
**male, n(%)**	715 (61.5)	172 (61.2)	312 (66.0)	484 (64.2)
**Primary renal disease, n(%)**				
Diabetes	103 (8.9)	49 (17.4)	72 (15.2)	121 (16.0)
Glomerulonephritis	266 (22.9)	47 (16.7)	72 (15.2)	119 (15.8)
Renal vascular disease	153 (13.2)	55 (19.60)	90 (19.0)	145 (19.2)
Cystic kidney disease	210 (18.1)	31 (11.0)	55 (11.6)	86 (11.4)
Other diseases	267 (22.9)	55 (19.5)	113 (23.9)	168 (22.7)
Unknown ontology	164 (14.1)	44 (15.7)	71 (15.0)	115 (15.3)
**Comorbidities, n(%)**^**a**^				
Cardiac event	105 (9.3)	40 (14.8)	72 (15.9)	112 (15.5)
Vascular event	75 (6.6)	29 (10.7)	47 (10.5)	76 (10.6)
Cerebrovascular accident	50 (4.4)	25 (9.3)	41 (9.1)	66 (9.1)
Diabetes	220 (17.4)	71 (27.6)	112 (25.8)	183 (26.5)
**Median dialysis vintage (IQR), mo**	0 (0–12)	27 (14–43)	23 (12–40)	24 (13–40)
**Preemptive transplantation, n(%)**	666 (57.3)	35 (12.5)	64 (13.5)	99 (13.1)
**Mean BMI (SD), kg/m**^**2 a**^	26.4 (4.7)	26.3 (4.8)	26.4 (4.2)	26.3 (4.4)
**SES rank, n(%)**				
Low	252 (21.7)	103 (36.7)	154 (32.6)	257 (34.1)
Medium	764 (65.7)	150 (53.4)	275 (58.1)	425 (56.4)
High	147 (12.6)	28 (10.0)	44 (9.3)	72 (9.5)
**Donor related factors**				
Mean age (SD), yr	54.2 (11.7)	54.9 (15.2)	55.6 (14.0)	55.3 (14.5)
Male, n(%)	471 (40.5)	145 (51.6)	307 (64.9)	452 (59.9)
Mean BMI (SD), kg/m^2 a^	26.3 (3.6)	25.8 (4.0)	25.9 **(**4.9)	25.8 **(**4.6)
Hypertension, n(%) ^a^	15 (8.8)	91 (35.1)	142 (30.9)	233 (32.5)
Mean serum creatinine (SD), μmol/l	- ^b^	80.1 (41.9)	67.6 (26.1)	72.3 (33.5)
Extended criteria donor, n(%)^a^	–	159 (56.6)	242 (51.2)	401 (53.2)
**ABO incompatibility, n(%)**	82 (7.1)	0 (0)	0 (0)	0 (0)
**Transplant PRA, n(%)** ^**a**^				
< 5%	1083 (93.3)	265 (94.3)	453 (95.8)	718 (95.2)
5–85%	77 (6.6)	15 (5.3)	19 (4.0)	34 (4.5)
> 85	1 (0.1)	1 (0.4)	1 (0.4)	1 (0.4)
**HLA-ABDR mismatches, n(%)** ^**a**^				
0	15 (4.2)	37 (13.2)	19 (4.0)	56 (7.4)
1	20 (5.6)	8 (2.8)	30 (6.4)	38 (5.1)
2	43 (12.1)	51 (18.1)	117 (24.8)	168 (22.3)
3	89 (25.0)	95 (33.8)	145 (30.8)	240 (31.9)
4	85 (23.9)	51 (18.1)	96 (20.4)	147 (19.5)
5	74 (20.8)	28 (10.0)	50 (10.6)	78 (10.4)
6	30 (8.4)	11 (3.9)	14 (3.0)	25 (3.3)
**Mean WIT1 (SD), min** ^**a**^	–	–	15.4 (4.9)	–
**Mean WIT2 (SD), min** ^**a**^	28.5 (15.7)	34.6 (17.9)	32.1 (14.2)	33.0 (15.7)
**Mean CIT (SD), hr** ^**a**^	2.2 (0.6)	12.9 (4.8)	11.9 (4.3)	12.2 (4.5)
**DGF, n(%)**	25 (0.02)	55 (19.6)	218 (46.1%)	273 (36.2)

Data are presented as mean ± SD or median (IQR) for continuous variables, and as n (%) for categorical variables

a. Variables with missing values. In the LDKT group: cardiac event (2.9%), vascular vent (2.5%), cerebral vascular accident (2.6%), diabetes (6.9%), BMI (71.6%), donor-related factor (BMI, 7.0% and hypertension, 85.4%), transplant PRA (0.2%), mismatch ABDR (69.4%), WIT2 (25.3%), CIT (43.3%). In the DDKT group: cardiac event (4.0%), vascular vent (4.8%), cerebral vascular accident (4.2%), diabetes (8.4%), BMI (63.9%), donor-related factor (hypertension, 4.8% and extended criteria deceased donor, 1.7%), HLA-ABDR mismatch (0.3%), WIT1(8.5%, only in DCD group), WIT2 (10.5%), CIT (12.2%)

b. The serum creatinine level was not collected by the registry and was assumed normal in all living donors

**Table 2:** Distribution of 3-month hospital readmission in each patient group (*n* = 1917)

Patient group	Hospital readmission = 0 (***n*** = 941, 49.1%)	Hospital readmission ≥ 1 (***n*** = 615, 32.1%)	Hospital readmission (missing) (***n*** = 361, 18.8%)
LDKT recipients, n(%)			
< 65 yr	447 (49.4)	253 (28.0)	204 (22.6)
≥ 65 yr	116 (44.8)	86 (33.2)	57 (22.0)
Total	563 (48.4)	339 (29.1)	261 (22.4)
DDKT recipients, n(%)			
< 65 yr	218 (49.5)	163 (37.0)	59 (13.4)
≥ 65 yr	160 (51.0)	113 (36.0)	41 (13.1)
Total	378 (50.1)	276 (36.6)	100 (13.3)
DDKT recipients with a DBD donor, n(%)			
< 65 yr	77 (48.4)	57 (35.8)	25 (15.7)
≥ 65 yr	59 (48.4)	47 (38.5)	16 (13.1)
Total	136 (48.4)	104 (37.0)	41 (14.6)
DDKT recipients with a DCD donor, n(%)			
< 65 yr	141 (50.2)	106 (37.7)	34 (12.1)
≥ 65 yr	101 (52.6)	66 (34.4)	25 (13.0)
Total	242 (51.2)	172 (36.4)	59 (12.5)

Sensitivity analyses were conducted to test the robustness of our results. First, all analyses were repeating using a complete case approach (using only patients without missing data). Second, we repeated the analyses with age as a continuous variable in the model to investigate whether the relationship between donor type and hospital readmission was modified by age. Finally, we conducted the analysis while controlling for all potential baseline confounders (Figure S1-2) presented in the DAGs instead of only the minimum set of confounders. ORs were reported with 95% confidence intervals (CI). *P* ≤ 0.05 (two-tailed) was considered statistically significant. All analyses were performed with SPSS software version 25.0. (IBM, Armonk, NY, USA).

## Results

In total, 2660 patients underwent kidney transplantation between 1 January 2016 and 31 December 2018 in The Netherlands. We excluded 256 patients from one medical centre due to a high proportion of missing value (87.9%) in the study outcome (for characteristics of excluded KTRs, see **Table** **S2**). Hereafter, a total of 1988 first time KTRs were identified, of whom 1942 were considered eligible for hospital readmission as they survived more than 14 days without the diagnosis of graft failure. Finally, we excluded 25 cases with competing events (death or graft failure), leaving 1917 patients (72.1%) included in our analyses (Fig. 1).

### Baseline characteristics

Table 1 presents the demographic and clinical characteristics of KTRs and donors. Of the 1917 included patients, 1163 (60.7%) patients had LDKT and 754 (39.4%) patients had DDKT. Compared to DDKT, LDKT recipients had a younger age, better SES, shorter time on dialysis, a higher chance of preemptive transplantation, fewer comorbidities, and a higher level of HLA-ABDR mismatch. Due to the elective nature of LDKT, the cold ischemia time was considerably shorter in LDKT recipients than that in DDKT recipients. Compared to the deceased donors, the living donors were less likely to have higher serum creatinine levels and hypertension. Among DDKT recipients, 281 patients (37.2%) received kidneys from DBD donors and 473 patients (62.3%) received kidneys from DCD donors. Marginal differences concerning baseline characteristics were found between these two groups except for WIT1 as there is no WIT1 in kidney transplantation with DBD donors, and it is, on average, 15.4 mins in kidney transplantation with DCD donors. The prevalence of DGF in KTRs with different donor types varied considerably: 2% in patients with LDKT and 36.2% in patients with DDKT (of which: 19.6% in KTRs with DBD donors and 46.1% in KTRs with DCD donors).

### Outcome distribution in each KTR group (donor type and age)

The registry data showed that, within the first 3 months after kidney transplantation, 941 (49.1%) KTRs had no hospital readmission, 615 (32.1%) KTRs had at least one readmission, and 361 KTRs (18.8%) had missing values in the outcome. The number of hospital readmission ranged from 0 to 11 in all KTRs, and the median number (IQR) of hospital readmissions was 1 (1–2) in KTRs with at least one hospital readmission. Table 2 shows the hospital readmission distribution in different KTRs groups stratified by donor type and by age. The median number (IQR) of hospital readmissions in readmitted KTRs was 1 (1–2) in all subgroups.

### 3-month hospital readmission following kidney transplantation

The risk of post-transplant hospital readmission within the first 3 months was lower after LDKT compared to DDKT (adjusted OR (ORadj) = 0.76; 95%CI, 0.61 to 0.96; *p* = 0.02). In the young recipients, a lower risk of hospital readmission was found after LDKT compared to DDKT (ORadj = 0.69, 95%CI, 0.52 to 0.90, *p* = 0.01) but this difference in risk was not detected in the elderly recipients (ORadj = 0.93, 95%CI, 0.62 to 1.39, *p* = 0.73). The risk in the young did not differ significantly from that in the elderly (*p*-value for interaction = 0.38). Among DDKT recipients, the risk of 3-month hospital readmission was similar between KTRs with DBD and DCD donors. The young and elderly DDKT recipients also had a similar risk of hospital admission after adjusting for potential baseline confounders (Table 3).

**Table 3:** Multivariable logistic regression models for the association between donor type and post-transplant hospital readmission within 3 months (n = 1917)

KTRs	Crude OR	95% CI	*P* value	Adjusted OR	95% CI	P value
Living donor versus deceased donor (reference)^a, c^						
All age group	0.74	0.60–0.91	0.004	0.76	0.61–0.96	0.02
< 65 yr	0.68	0.53–0.88	0.003	0.69	0.52–0.90	0.01
≥ 65 yr	0.93	0.64–1.35	0.70	0.93	0.62–1.39	0.73
DCD donor versus DBD donor (reference)^b, d^						
All age group	0.93	0.67–1.28	0.64	0.89	0.60–1.32	0.57
< 65 yr	1.02	0.66–1.57	0.91	0.90	0.54–1.49	0.67
≥ 65 yr	0.81	0.50–1.31	0.38	0.88	0.47–1.67	0.69

a. Variables adjusted in the all age group included recipient age (continuous), recipient sex, primary disease, comorbidities, SES, PRA, and medical center. For a specific age group, all the above variables except for recipient age were included. In this comparison, transplantation with a deceased donor was used as a reference

b. Variables adjusted in the all age group included recipient age (continuous), primary disease, comorbidities, dialysis vintage, PRA, HLA-ABDR mismatch, donor characteristics (age, sex, BMI, hypertension, last serum creatinine before donation, and extended criteria deceased donor), and medical center. For a specific age group, all the above variables except for recipient age were included. In this comparison, transplantation with a DBD donor was used as a reference

c. P-value for interaction between age and donor type (living donor versus deceased donor): 0.38

d. *P*-value for interaction between age and donor type (DCD donor versus DBD donor): 0.54

### Sensitivity analyses

Compared to KTRs without missing values in hospital readmission, those with missing values were younger, more likely to be LDKT recipients and thus, had shorter dialysis vintage or preemptive transplantation, and were less likely to be DDKT recipients with DCD donor **(Supplementary Table** **S1****)**. The complete case analysis yielded qualitatively similar findings compared to the main analysis, except for the unadjusted OR between LDKT and DDKT recipients in the all age group, potentially due to bias and loss of power introduced by the missing values (OR = 0.83; 95%CI, 0.67 to 1.01; *p* = 0.07) (**Supplementary Table** **S3**). When repeating the analysis with age as a continuous variable in the model, similar results were found: the modification effect by age was not significant (LDKT vs DDKT: *p*-value for interaction term = 0.79; DBD donors vs DCD donors within the DDKT group: *p-*value for interaction = 0.48). When adjusting for all potential baseline confounders presented in the DAG,, the results (**Supplementary Table** **S4**) were in line with that of the main analysis in which only a minimum set of confounders was included (Table 3).

## Discussion

This registry study investigated post-transplant 3-month hospital readmission in KTRs with different donor types. The results suggested that, in the overall population, LDKT recipients had a lower risk of hospital readmissions during the first 3 months after kidney transplantation compared to DDKT recipients. In the young recipients, the risk of hospital readmission after LDKT compared DDKT was lower but did not significantly differ from that in the elderly. Within the DDKT group, KTRs with DBD and DCD donors had a similar risk of hospital readmission, regardless of age.

To the best of our knowledge, to date, only a few studies have shed light on early hospital readmission after kidney transplantation and its association with donor type. McAdams-Demarco and colleagues reported a higher risk of 30-day hospital readmission in KTRs with either DCD donors or expanded criteria donors (i.e. a deceased donor over 60 years old or a deceased donor over 50 years old with two of the following risk actors: hypertension, high serum creatinine and death due to stoke) as compared to those with living donors [2]. Boubaker and colleagues also described a higher readmission rate in DDKT recipients than that in LDKT recipients [11]. The results of our study are in line with and add to findings from previous research by a stepwise comparison of different donor types –comparing LDKT and DDKT at first and then comparing DDKT with DBD and DCD donors in the Netherlands –while also exploring the modification effect of age. The latter is of great clinical interest due to the shift to an older age in kidney transplant recipients in the past decades.

The lower risk of 3-month hospital readmission in LDKT recipients compared to DDKT recipients may have different ontologies. First, LDKT recipients are more likely to have preemptive transplantation or a shorter dialysis vintage and are therefore less likely to experience early hospital readmission as they are in relatively better health compared to DDKT recipients [1, 22]. In our study, DDKT recipients had on average a 24 months longer dialysis vintage and less often underwent preemptive transplantation (12.5% in DDKT recipients and 57.3% in LDKT recipients) than LDKT recipients. Second, the quality of the donor kidney from living donors is superior compared to deceased donors because living donors should meet eligibility requirements related to their health status [23]. Indeed, living donors in our study were less likely to have hypertension and abnormal renal function compared to deceased donors. Previous studies also reported an association of donor characteristics with delayed graft function (DGF), which could be the primary reason for early hospital readmission [9, 24]. Third, the transplantation process is different between LDKT and DDKT. In our study, the average CIT of LDKT was 10 h shorter compared to DDKT due to its elective nature (2.2 versus 12.2 h). This prolonged CIT is a risk factor for delayed graft function, hospital readmission, and a prolonged hospital stay for transplantation [25]. In our study population, only 2% of the LDKT recipients experienced DGF while 36.2% in DDKT recipients.

Furthermore, a significantly lower risk of 3-month hospital readmission after LDKT compared to DDKT was found in the young recipients, but the risk was comparable in the elderly recipients. It is important to note that, although the modification effect by age on a multiplicative scale was not statistically significant, there are indications of a modification effect. The difference between the ORs (point estimate) in the young and elderly implied potentially attenuated benefit of LDKT in the elderly. Notably, the statistical insignificance of the interaction term could be a result of the relatively small sample size of elderly KTRs included in the analysis. A possible explanation for this potential modification effect of age could be that comorbidities and frailty in elderly patients may offset the potential benefits of LDKT. Elderly patients are more likely to be comorbid compared to their younger counterparts at the time of kidney transplantation (48.2% in elderly KTRs and 28.9% in young patients) [26], and frailty is also highly prevalent in elderly patients receiving renal replacement therapy [27]. The explanation is furthermore supported by a study suggesting that frailty at the time of kidney transplantation – evaluated as unintentional weight loss, weakness, exhaustion, low activity, and slowed walking speed – independently predicts a 60% higher risk of readmission [28].

Finally, our study suggested comparability in the risk of 3-month hospital readmission in KTRs with DBD and DCD donors, regardless of age. Since KTRs with DCD donors have a reportedly higher incidence of DGF compared to those with DBD donors, [9] one might, therefore, expect a higher risk of hospital readmission in KTRs with DCD donors. A possible explanation for the absence of this relationship is that the difference in hospital readmission caused by DGF becomes negligible due to other potential causes for hospital readmission in these two groups who share similar demographic and clinical characteristics (e.g., older age, more comorbidities, and longer CIT).

This registry study has several strengths. To the best of our knowledge, this is the first study to investigate the effect of donor type on hospital readmission in Dutch KTRs. By using data of a Dutch national registry, this study was conducted with a more representative sample of the KTR population in the Netherlands compared to single-centre studies. Furthermore, controlling confounding by a selection of confounders identified by the DAG approach, instead of adding all clinically relevant variables into the models, can provide more robust estimates for an etiological purpose. Nevertheless, awareness of the limitations is necessary when interpreting the results. First, the exclusion of one medical centre due to a high proportion of missing values might marginally influence the generalization of our study results **(Table** **S2****)**. Second, being an observational study, this study is potentially limited by residual confounding. Third, the sample size of elderly KTRs might lead to a wide confidence interval and less precise results for the subgroup analysis. Finally, the cause of hospital readmission was not available in the registry. Therefore, we could not distinguish between the planned and unplanned hospital readmission. Also, without the information regarding the date of hospital readmission and the length of hospital stay, there is a lack of in-depth understanding of the hospital readmission. The generalizability of our findings to other populations or countries depends on the demographic and clinical characteristics of KTRs as well as the health care delivery systems. In the Netherlands, the medical system is based on mandatory health insurance with universal health care coverage [29], and there are no financial penalties for a hospital due to hospital readmissions. Therefore, the hospital readmission reflects the need for care without financial concerns from patients and hospitals.

Hospital (re) admissions, planned or unplanned, can reflect the disease burden in KTRs [6]. According to a national registry study in the U.S., the median length of hospital stay for the early readmission after kidney transplantation reached 9 days (IQR, 4 to 16) [30]. The findings of our study suggest that DDKT recipients have a higher short-term disease burden due to a higher risk of 3-month hospital readmission compared to LDKT recipients, and the difference might not be dominant in elderly KTRs. DDKT recipients with DBD or DCD donors have a similar level of diseased burden reflected by hospital readmission. Despite that the donor type is hardly modifiable, this information allows healthcare professionals to identify high-risk patients and consequently prevent unnecessary hospital readmission. The leading causes for early post-transplant hospital readmission in KTRs are graft rejection, infection, post-surgical complications, abnormal graft function, volume overloaded or depletion, infection, drug toxicity, disturbed electrolytes, and anaemia [12, 24]. Previous research has shown that interventions by healthcare professionals at different levels can reduce unnecessary hospital readmission [12, 22, 31]. For example, patient education about post-transplant medication, especially immunosuppressants, can improve medication adherence in KTRs and therefore, prevent graft rejection [22]. Other interventions to reduce readmission include medication reconciliation, discharge planning before discharge, and timely communication and follow-up [31]. From the patients’ perspective, better preparation and planning before discharge, clarity in discharge instructions, promotion of the resources in outpatient clinics, and aligned attitude and expectations of patients and healthcare professionals were identified as essential aspects to reduce readmission [32]. Notably, it is not always true that the fewer hospital readmissions, the better. Post-transplant readmission may be necessary to achieve better health for patients with poor understanding or a lack of regular outpatient check-ups due to intellectual and financial challenges [32, 33].

## Conclusions

In conclusion, the risk of 3-month hospital readmission is lower in LDKT recipients compared to DDKT recipients and this benefit of LDKT might be less dominant in elderly KTRs. In DDKT, recipients with DCD and DBD donors had a similar risk of 3-month hospital readmission in the overall population and in the separated age groups. Therefore, greater attention and tailored patient management is necessary to reduce unnecessary hospital readmission in elderly KTRs and KTRs with DDKT.

## Supplementary Information

**Additional file 1 Figure S1**. DAG for the effect of donor type on post-transplant 3-month hospital readmission: living donor kidney transplantation versus deceased donor kidney transplantation.

**Additional file 2 Figure S2**. DAG for the effect of donor type on post-transplant 3-month hospital readmission: kidney transplantation with donation after cardiac death (DCD) versus donation after brain death (DBD).

**Additional file 3 Table S1**. Demographic and clinical characteristics between kidney transplant recipients with and without missing values in the outcome (*n* = 1917).

**Additional file 4 Table S2.** Demographic and clinical characteristics between KTRs included in the analysis and the eligible KTRs from the excluded medical centre.

**Additional file 5 Table S3**. Multivariate logistic regression models for the association between donor type and post-transplant hospital readmission within 3 months: a complete case analysis (n = 1556).

**Additional file 6 Table S4.** Multivariable logistic regression models for the association between donor type and post-transplant hospital readmission within 3 months with all confounders (n=1917).

## Data Availability

The de-identified data underlying this article were provided by the Netherland Organ Transplantation Registry (NOTR). Data are available with the permission of NOTR (https://www.transplantatiestichting.nl/).

## Abbreviations

BMI: Body mass index
CI: Confidence interval
CIT: Cold ischemia time
DAG: Directed acyclic graph
DBD: Donation after brain death
DCD: Donation after cardiac death
DDKT: Deceased donor kidney transplantation
DGF: Delayed graft function
HLA-ABDR mismatch: human leucocyte antigen-ABDR mismatch
IQR: Interquartile range
KTRs: Kidney transplant recipients
LDKT: Living donor kidney transplantation
OR: Odds ratio
ORadj: Adjusted odds ratio
PRA: Panel-reactive antibody
SD: Standard deviation
SES: Socioeconomic status
WIT1: The first warm ischemia time
WIT2: The second warm ischemia time
