# Supplementary material for: Donor type and 3-month hospital readmission following kidney transplantation: results from the Netherlands organ transplant registry
Source: BMC Nephrol. 2021 Apr 27;22:155. doi: 10.1186/s12882-021-02363-5 (PMC8077946; doi:10.1186/s12882-021-02363-5)
Supplement: Supplementary file 1 — Additional file 1 Figure S1. DAG for the effect of donor type on post-transplant 3-month hospital readmission: living donor kidney transplantation versus deceased donor kidney transplantation. [file 12882_2021_2363_MOESM1_ESM.docx]

**
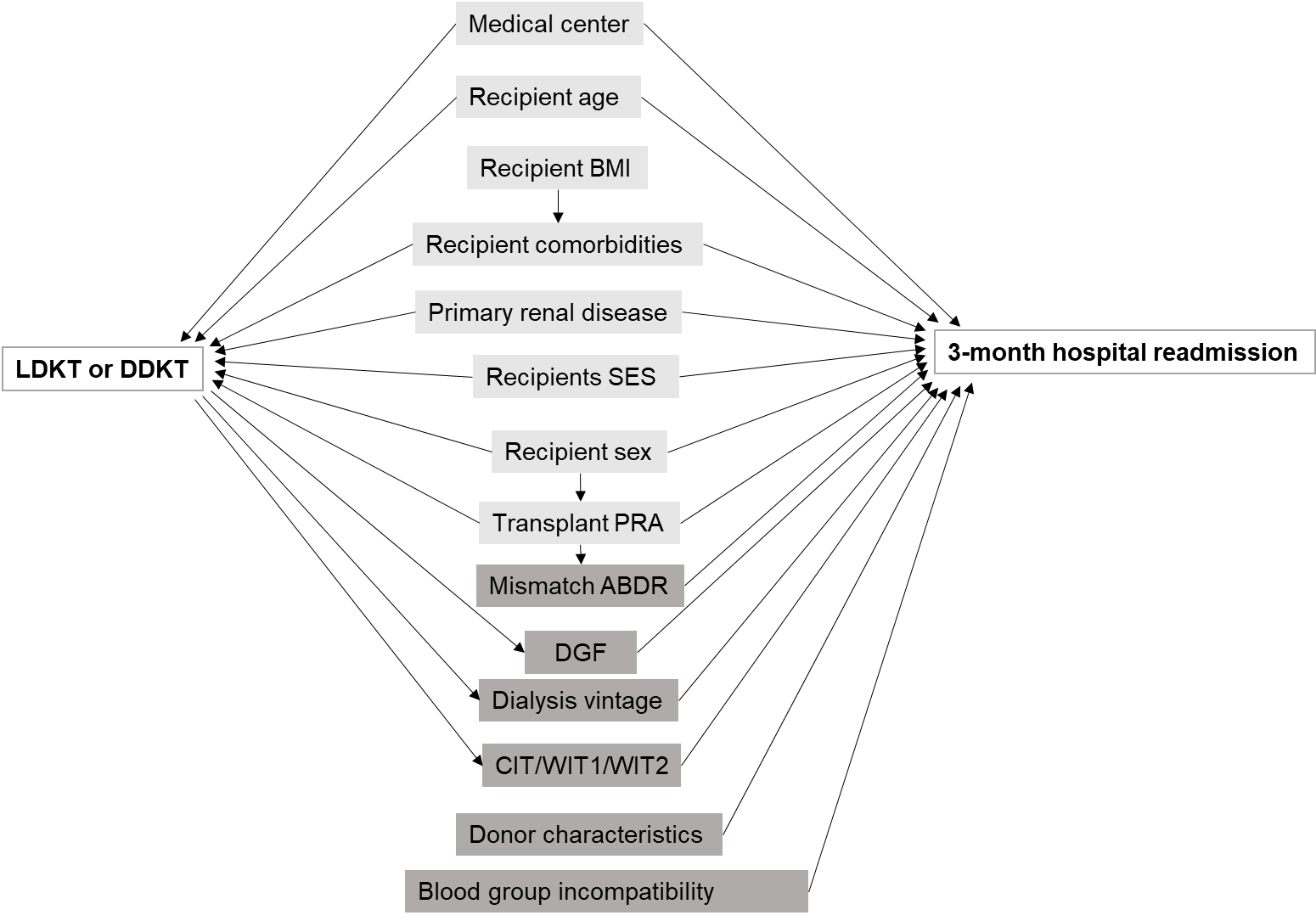
**

**Additional file 1: Figure S1**. DAG for the effect of donor type on post-transplant 3-month hospital readmission: living donor kidney transplantation versus deceased donor kidney transplantation.

Donor characteristics include donor age, sex, BMI, history of hypertension, and the last serum creatinine level before donation. Variables in the light grey boxes were identified as potential cofounders. According to the DAGs approach, it is sufficient to adjust for one confounder when there are more than one on the same path (e.g. when the recipient comorbidities are adjusted in the model, it is not necessary to adjust for recipient BMI) (Pearl, 1993). It should be noted that the variable DGF was not classified as a confounder but as a mediator which mediates the impact of donor type on hospital readmission.
