# Supplementary material for: Donor type and 3-month hospital readmission following kidney transplantation: results from the Netherlands organ transplant registry
Source: BMC Nephrol. 2021 Apr 27;22:155. doi: 10.1186/s12882-021-02363-5 (PMC8077946; doi:10.1186/s12882-021-02363-5)
Supplement: Supplementary file 2 — Additional file 2 Figure S2. DAG for the effect of donor type on post-transplant 3-month hospital readmission: kidney transplantation with donation after cardiac death (DCD) versus donation after brain death (DBD). [file 12882_2021_2363_MOESM2_ESM.docx]

**
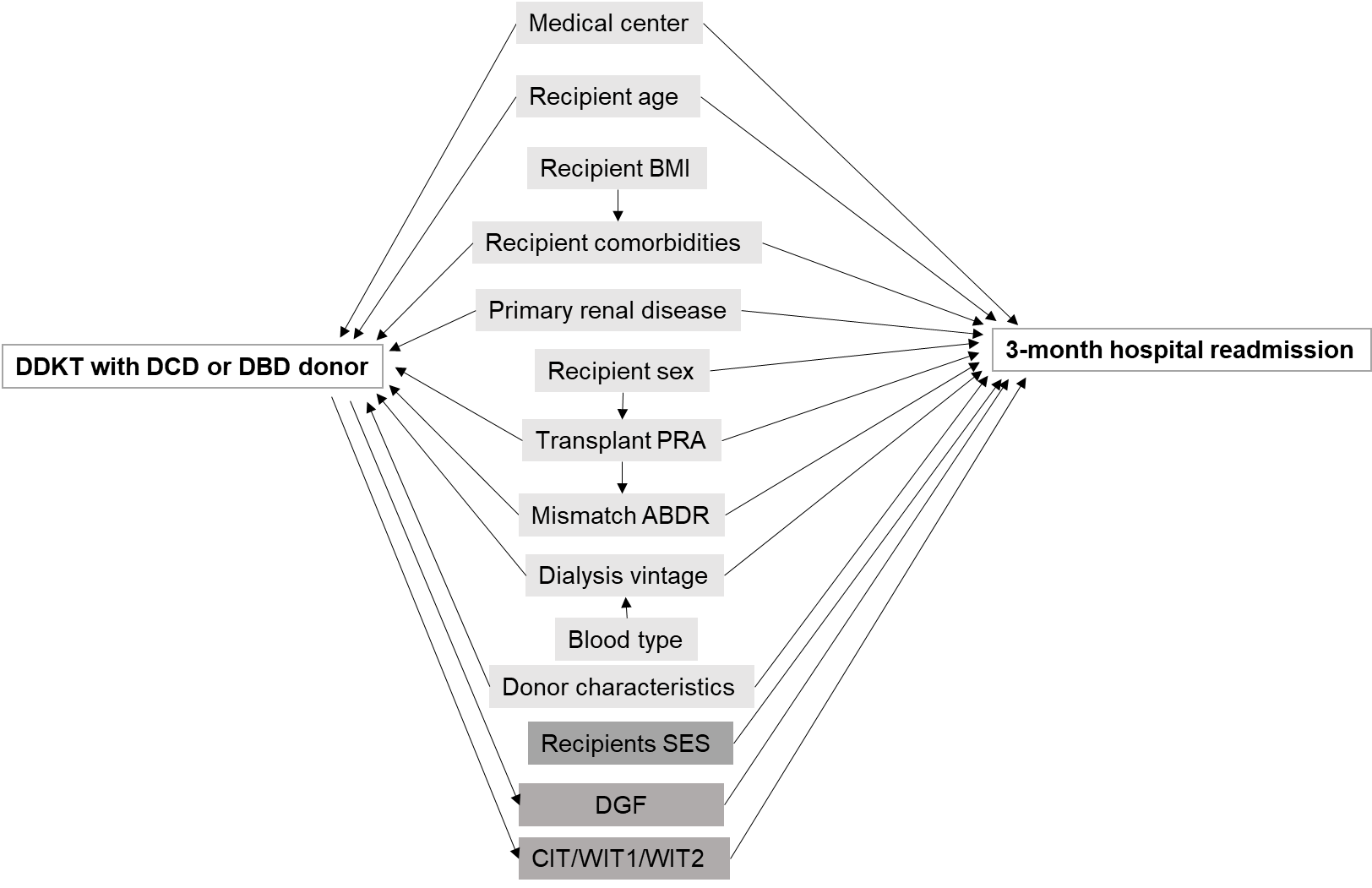
**

**Additional file 2: Figure S2**. DAG for the effect of donor type on post-transplant 3-month hospital readmission: kidney transplantation with donation after cardiac death (DCD) versus donation after brain death (DBD).

Donor characteristics include donor age, sex, BMI (body mass index), history of hypertension, the last serum creatinine level before donation, and extended criteria deceased donor. Variables in the light grey boxes were identified as potential cofounders. Following the same argument in the footnote of Figure S1., it is not necessary to put recipient BMI, recipient sex, and blood type into the model. It should be noted that the variable DGF was not classified as a confounder but as a mediator which mediates the impact of donor type on hospital readmission.
