# Supplementary material for: Donor type and 3-month hospital readmission following kidney transplantation: results from the Netherlands organ transplant registry
Source: BMC Nephrol. 2021 Apr 27;22:155. doi: 10.1186/s12882-021-02363-5 (PMC8077946; doi:10.1186/s12882-021-02363-5)
Supplement: Supplementary file 3 — Additional file 3 Table S1. Demographic and clinical characteristics between kidney transplant recipients with and without missing values in the outcome (n = 1917). [file 12882_2021_2363_MOESM3_ESM.docx]

**Additional file 3: Table S1**. Demographic and clinical characteristics between kidney transplant recipients with and without missing values in the outcome (n=1917)

| **Characteristics** | **KTRs without missing values in the outcome** | **KTRs with missing values in the outcome** |
| --- | --- | --- |
| **N(%)** | 1556 (81.2) | 361 (18.8) |
| **Age category, yr, n(%)** |  |  |
| 18~39 | 220 (14.1) | 68 (18.8) |
| 40~59 | 614 (39.5) | 145 (40.2) |
| 60~64 | 247 (15.9) | 50 (13.9) |
| 65~ | 475 (30.5) | 98 (27.1) |
| **Male, n(%)** | 979 (62.3) | 220 (60.1) |
| **SES rank, n(%)** |  |  |
| Low | 410 (26.3) | 99 (27.4) |
| Medium | 961 (61.8) | 228 (63.2) |
| High | 185 (11.9) | 34 (9.4) |
| **LDKT, n(%)** | 902 (60.0) | 261 (72.3) |
| **DKKT, n(%)** |  |  |
| With DBD donors | 240 (15.4) | 41 (11.4) |
| With DCD donors | 414 (26.6) | 59 (16.3) |
| **Primary renal disease, n(%)** |  |  |
| Diabetes | 201 (12.9) | 23 (6.4) |
| Glomerulonephritis | 316 (20.3) | 69 (19.1) |
| Renal vascular disease | 245 (15.7) | 53 (14.7) |
| Cystic kidney disease | 232 (14.9) | 64 (17.7) |
| Other diseases | 357 (22.9) | 78 (21.6) |
| Unknown ontology | 205 (13.2) | 74 (20.5) |
| **Comorbidities, n(%)*** |  |  |
| Cardiac event | 193 (12.8) | 24 (6.9) |
| Vascular event | 124 (8.2) | 27 (7.8) |
| Cerebral vascular accident | 102 (6.8) | 14 (4.1) |
| Diabetes | 310 (21.5) | 43 (13.1) |
| **Median dialysis vintage(IQR), mo** | 10 (0-26) | 5 (0-23) |
| **Preemptive transplantation, n(%)** | 608 (39.1) | 157 (43.5) |
| **Mean BMI (SD), kg/m^2^*** | 26.5 (4.6) | 25.8 (4.6) |

*Variables with missing values. In KTRs without missing values in outcome: cardiac event (3.0%), vascular vent (3.1%), cerebral vascular accident (3.0%), diabetes (7.1%) and BMI (66.3%). In KTRs without missing values in outcome: cardiac event (4.2%), vascular vent (4.4%), cerebral vascular accident (4.4%), diabetes (8.9%) and BMI (65.9%).
