# Supplementary material for: Donor type and 3-month hospital readmission following kidney transplantation: results from the Netherlands organ transplant registry
Source: BMC Nephrol. 2021 Apr 27;22:155. doi: 10.1186/s12882-021-02363-5 (PMC8077946; doi:10.1186/s12882-021-02363-5)
Supplement: Supplementary file 4 — Additional file 4 Table S2. Demographic and clinical characteristics between KTRs included in the analysis and the eligible KTRs from the excluded medical centre. [file 12882_2021_2363_MOESM4_ESM.docx]

**Additional file 4: Table S2.** Demographic and clinical characteristics between KTRs included in the analysis and the eligible KTRs from the excluded medical centre

| **Characteristics** | **KTRs included in the analysis**  **(n=1917)** | **Eligible KTRs excluded from one medical centre (n=247^a^)** |
| --- | --- | --- |
| **Age category, yr, n(%)** |  |  |
| 18~39 | 288 (15.0) | 36 (14.6) |
| 40~59 | 759 (39.6) | 93 (37.7) |
| 60~64 | 297 (15.5) | 49 (19.8) |
| 65~ | 573 (29.9) | 69 (27.9) |
| **Male, n(%)** | 1199 (62.5) | 158 (64.0) |
| **SES rank, n(%)** |  |  |
| Low | 509 (26.6) | 63 (25.5) |
| Medium | 1189 (62.0) | 150 (60.7) |
| High | 219 (11.4) | 34 (13.8) |
| **LDKT, n(%)** | 1163 (60.7) | 114 (46.2) |
| **DKKT, n(%)** |  |  |
| With DBD donors | 281 (14.7) | 47 (19.0) |
| With DCD donors | 473 (24.7) | 86 (34.8) |
| **Primary renal disease, n(%)** |  |  |
| Diabetes | 224 (11.7) | 29 (11.7) |
| Glomerulonephritis | 385 (20.1) | 37 (15.0) |
| Renal vascular disease | 298 (15.5) | 48 (19.4) |
| Cystic kidney disease | 296 (15.4) | 42 (17.0) |
| Other diseases | 346 (18.0) | 32 (13.0) |
| Unknown ontology | 279 (14.6) | 45 (18.0) |
| **Comorbidities, n(%)*** |  |  |
| Cardiac event | 217 (11.3) | 27 (10.9) |
| Vascular event | 151 (7.9) | 26 (10.5) |
| Cerebral vascular accident | 116 (6.1) | 15 (6.1) |
| Diabetes | 54 (21.9) | 54 (21.9) |
| **Median dialysis vintage(IQR), mo** | 9 (0-26) | 14 (0-34) |
| **Preemptive transplantation, n(%)** | 765 (39.9) | 103 (41.7) |
| **Mean BMI (SD), kg/m^2^*** | 26.4 (4.6) | 26.2 (4.8) |

a. among 256 KTRs excluded from one medical centre due to a high percentage of missing values in outcome (87.9%), 247 met the eligibility of our analysis.

*Variables with missing values. In KTRs included in the analysis: cardiac event (3.3%), vascular vent (3.4%), cerebral vascular accident (3.2%), diabetes (7.5%) and BMI (68.6%). In eligible KTRs excluded from one medical centre: and BMI (71.7%).
