# Supplementary material for: Donor type and 3-month hospital readmission following kidney transplantation: results from the Netherlands organ transplant registry
Source: BMC Nephrol. 2021 Apr 27;22:155. doi: 10.1186/s12882-021-02363-5 (PMC8077946; doi:10.1186/s12882-021-02363-5)
Supplement: Supplementary file 5 — Additional file 5 Table S3. Multivariate logistic regression models for the association between donor type and post-transplant hospital readmission within 3 months: a complete case analysis (n = 1556). [file 12882_2021_2363_MOESM5_ESM.docx]

**Additional file 5: Table S3**. Multivariate logistic regression models for the association between donor type and post-transplant hospital readmission within 3 months: a complete case analysis (n=1556)

| KTRs | Crude OR | 95% CI | P value | Adjusted OR | 95% CI | P value |
| --- | --- | --- | --- | --- | --- | --- |
| Living donor versus deceased donor (reference) ^a, c^ | | | | | | |
| All age group | 0.83 | 0.67-1.01 | 0.07 | 0.78 | 0.62-0.98 | 0.03 |
| < 65 yr | 0.76 | 0.59-0.98 | 0.03 | 0.72 | 0.54-0.95 | 0.02 |
| ≥ 65 yr | 1.05 | 0.73-1.52 | 0.80 | 0.98 | 0.65-1.48 | 0.92 |
| DCD donor versus DBD donor (reference) ^b, d^ | | | | | | |
| All age group | 0.93 | 0.67-1.28 | 0.66 | 0.97 | 0.65-1.42 | 0.87 |
| < 65 yr | 1.02 | 0.66-1.55 | 0.94 | 1.01 | 0.59-1.73 | 0.99 |
| ≥ 65 yr | 0.82 | 0.50-1.34 | 0.43 | 1.25 | 0.63-2.49 | 0.53 |

a. Variables adjusted in the all age group included recipient age (continuous), recipient sex, primary disease, comorbidities, SES, PRA, and medical centre. For a specific age group, all the above variables except for recipient age were included. In this comparison, transplantation with a deceased donor was used as a reference.

b. Variables adjusted in the all age group included recipient age (continuous), primary disease, comorbidities, dialysis vintage, PRA, HLA-ABDR mismatch, donor characteristics (age, sex, BMI, hypertension, last serum creatinine before donation, and extended criteria deceased donor), and medical centre. For a specific age group, all the above variables except for recipient age were included. In this comparison, transplantation with a DBD donor was used as a reference.

c. P-value for interaction between age and donor type (living donor versus deceased donor): 0.25

d. P-value for interaction between age and donor type (DCD donor versus DBD donor): 0.67
