# Supplementary material for: Donor type and 3-month hospital readmission following kidney transplantation: results from the Netherlands organ transplant registry
Source: BMC Nephrol. 2021 Apr 27;22:155. doi: 10.1186/s12882-021-02363-5 (PMC8077946; doi:10.1186/s12882-021-02363-5)
Supplement: Supplementary file 6 — Additional file 6 Table S4. Multivariable logistic regression models for the association between donor type and post-transplant hospital readmission within 3 months with all confounders (n=1917). [file 12882_2021_2363_MOESM6_ESM.docx]

**Additional file 6: Table S4.** . Multivariable logistic regression models for the association between donor type and post-transplant hospital readmission within 3 months with all confounders (n=1917).

| KTRs | Adjusted OR | 95% CI | P value |
| --- | --- | --- | --- |
| Living donor versus deceased donor (reference)^a, c^ | | | |
| All age group | 0.78 | 0.63-0.96 | 0.02 |
| < 65 yr | 0.69 | 0.53-0.89 | 0.004 |
| ≥ 65 yr | 0.93 | 0.61-1.40 | 0.71 |
| DCD donor versus DBD donor (reference)^b, d^ | | | |
| All age group | 0.85 | 0.59-1.23 | 0.39 |
| < 65 yr | 0.85 | 0.53-1.37 | 0.51 |
| ≥ 65 yr | 0.86 | 0.45-1.65 | 0.67 |

a. Variables adjusted in the all age group included recipient age (continuous), recipient sex, recipient BMI, primary disease, comorbidities, SES, PRA, and medical center. For a specific age group, all the above variables except for recipient age were included. In this comparison, transplantation with a deceased donor was used as a reference.

b. Variables adjusted in the all age group included recipient age (continuous), recipient sex, recipient BMI, recipient blood type, primary disease, comorbidities, dialysis vintage, PRA, HLA-ABDR mismatch, donor characteristics (age, sex, BMI, hypertension, last serum creatinine before donation, and extended criteria deceased donor), and medical center. For a specific age group, all the above variables except for recipient age were included. In this comparison, transplantation with a DBD donor was used as a reference.

c. P-value for interaction between age and donor type (living donor versus deceased donor): 0.35

d. P-value for interaction between age and donor type (DCD donor versus DBD donor): 0.56
